# Identifying Opponent’s Neuroticism Based on Behavior in Wargame

**DOI:** 10.3390/bs15081012

**Published:** 2025-07-25

**Authors:** Sihui Ge, Sihua Lyu, Yazheng Di, Yue Su, Qian Luo, Aizhu Mei, Tingshao Zhu

**Affiliations:** 1 Institute of Psychology, Chinese Academy of Sciences, Beijing 100101, China; gesh@psych.ac.cn (S.G.); shlyu@163.com (S.L.); diyazheng97@163.com (Y.D.); ssssy0215@gmail.com (Y.S.); luoq@psych.ac.cn (Q.L.); meiaz@psych.ac.cn (A.M.); 2 Department of Psychology, University of Chinese Academy of Sciences, Beijing 100101, China

**Keywords:** wargame, neuroticism, machine learning, game behavior

## Abstract

Traditional neuroticism assessments primarily rely on self-report questionnaires, which can be difficult to implement in highly confrontational scenarios and are susceptible to subjective biases. To overcome these limitations, this study develops a machine learning-based approach using behavioral data to predict an opponent’s neuroticism in competitive environments. We analyzed behavioral records from 167 participants on the MiaoSuan Wargame platform. After data cleaning and feature selection, key behavioral features associated with neuroticism were identified, and predictive models were developed. Neuroticism was assessed using the 8-item neuroticism subscale of the Big Five Inventory. Results indicate that this method can effectively infer an individual’s neuroticism level. The best-performing model was LinearSVR, which balances interpretability, robustness to noise, and the ability to capture moderate nonlinear relationships—making it suitable for behavior-based psychological inference tasks. The correlation between predicted scores and self-reported questionnaire scores was 0.606, the R-squared value was 0.354, and the test–retest reliability was 0.516. These behavioral features provide valuable insights into neuroticism prediction and have practical applications in psychological assessment, particularly in competitive environments where conventional methods are impractical. This study demonstrates the feasibility of behavior-based neuroticism assessment and suggests future research directions, including refining feature selection techniques and expanding the application scenarios.

## 1. Introduction

In high-risk, highly adversarial environments such as military conflicts, success often depends on the ability to anticipate an opponent’s decisions under conditions of uncertainty and time pressure. These decisions are not always entirely rational; rather, they are frequently shaped by individual psychological traits, particularly the neuroticism aspect of personality (Boyes & French, 2010; Mendes et al., 2019). Research has shown that individuals with high neuroticism—that is, individuals whose behavior reflects heightened emotional instability in adversarial scenarios—tend to make erratic decisions. Additionally, they display heightened risk aversion when confronted with complex situations. In contrast, those with low neuroticism are generally more rational, calm, and stable, allowing them to make more consistent and calculated decisions under stress (Byrne et al., 2015; Lauriola et al., 2014). Consequently, assessing an opponent’s level of neuroticism can offer valuable insights into their behavioral tendencies, providing opportunities to exploit their psychological traits and design more effective counterstrategies. However, identifying an opponent’s neuroticism in real-time confrontations remains a critical and challenging task.

Studies have shown that high levels of stress significantly disrupt cognitive processing, which increases the likelihood of biased decision-making and errors in enemy identification (Gamble et al., 2018; Sekel et al., 2023). Recent reviews have further emphasized that acute psychological stress under uncertainty can impair risk-based decision-making through its interaction with individual personality traits—particularly neuroticism, which exacerbates stress sensitivity and reduces cognitive flexibility (de-Juan-Ripoll et al., 2021). These findings support our emphasis on understanding how neuroticism modulates behavioral tendencies in adversarial environments. In battlefield environments, high-pressure conditions impair cognitive functions. This makes individuals more prone to misidentifying friendly forces as adversaries, severely disrupting tactical execution and operational deployment. Similarly, an adversary’s psychological traits, such as neuroticism, can affect their ability to process information and make decisions under stress. Individuals with higher neuroticism are more likely to exhibit anxiety, impulsivity, and defensive behaviors, making them particularly susceptible to misinformation and psychological manipulation. Therefore, accurately assessing an adversary’s level of neuroticism is essential for anticipating their battlefield decisions. It also facilitates the development of effective deception strategies and psychological warfare tactics. Identifying an opponent’s neuroticism could improve judgment accuracy and strategic adaptability, allowing intelligence analysts to anticipate behavioral tendencies and potentially gain a competitive advantage in high-risk environments.

The absence of an effective method for assessing an opponent’s neuroticism presents significant challenges in adversarial decision-making. First, in competitive or hostile situations, individuals often conceal their psychological traits to avoid being strategically exploited (Dreu & Gross, 2019). Second, even when self-report data are gathered, participants frequently manipulate their responses to conform to social expectations, leading to biased results (Althubaiti, 2016; Cipresso & Riva, 2016; Montag et al., 2022). Furthermore, traditional personality assessments are typically conducted in controlled environments, which lack ecological validity, especially under high-pressure conditions. Therefore, there is an urgent need for innovative methods that can accurately assess an opponent’s neuroticism without relying on self-report.

Prior research has suggested that decision-making behavior in adversarial settings may reflect underlying personality traits, such as neuroticism. For example, individuals high in neuroticism have been associated with impulsive or stress-reactive strategies (Byrne et al., 2015), while emotionally stable individuals tend to adopt more cautious and rational planning (Mount et al., 1994; Komarraju et al., 2011). These behavioral tendencies may manifest in wargame scenarios through unit preferences and tactical decisions—for instance, favoring aggressive units like heavy tanks versus conservative units like infantry or artillery. Rather than relying on players’ conscious recognition of these psychological traits, this study investigates whether such behavioral regularities, when processed through machine learning algorithms, can reveal stable patterns that correspond to individual differences in neuroticism. These observations align with personality theory, which posits that neuroticism influences not only emotional reactivity but also behavioral coping strategies in competitive environments (Fréchette et al., 2017; Yılmaz & Kafadar, 2022). Thus, in high-stakes adversarial games, behavioral choices such as aggressive unit deployment or avoidance maneuvers may reflect deeper personality traits that are consistent and measurable.

Although the study is conceptually framed as inferring an opponent’s neuroticism—reflecting real-world scenarios where individuals must interpret the psychological states of adversaries based on behavior alone—the technical implementation remains strictly within-subject. Specifically, the models are trained to predict each individual’s self-reported neuroticism based on their own gameplay behavior. The term “opponent” is employed to emphasize the potential application in adversarial contexts, where psychological insights about others must be derived from indirect, observable indicators rather than direct assessments. This modeling choice is grounded in the principle that personality traits—especially neuroticism—manifest in consistent behavioral patterns under high-stakes conditions. In adversarial environments, behavioral cues such as risk aversion or response volatility can provide interpretable signals of underlying psychological traits. Our approach assumes that these behavioral indicators are reliable and generalizable enough to allow machine learning models trained on within-subject data to infer personality-related tendencies in others. This mirrors the logic of human attribution, where people routinely infer others’ internal dispositions based on observable actions (Jones & Davis, 1965).

In recent years, researchers have sought to integrate personality assessment with gaming behavior (Pereira et al., 2022). While prior studies have primarily focused on analyzing players’ own personality traits through self-reported data or in-game behavior, our study diverges by aiming to infer the neuroticism of one’s opponent in adversarial gaming contexts. However, current methods still have limitations. Some studies have examined the relationship between personality traits and gaming behavior using self-reported data (Holmgård et al., 2016; Phan & Rauthmann, 2021; Thue et al., 2007; Van Lankveld et al., 2011). Worth et al. found that players with different personality traits exhibit distinct behavioral patterns (Worth & Book, 2014). In My World, in-game behavior was shown to correlate with player motivation (Canossa et al., 2013), while in World of Warcraft, highly neurotic players tended to avoid conflict and exhibit decision instability (Yee et al., 2011). However, these studies rely on self-reported data, which are not applicable in confrontational scenarios. To address this limitation, recent research has employed immersive environments and behavioral data to assess individual traits without requiring explicit self-disclosure. For instance, a virtual-reality-based risk evaluation tool (AEMIN) has demonstrated that machine learning applied to behavioral metrics can effectively predict participants’ psychological risk profiles (including impulsivity and sensation seeking), providing a promising alternative to self-report measures (Shi et al., 2024). These advances highlight the value of ecologically valid, behavior-based assessment tools—an approach closely aligned with our proposed framework. Additionally, some studies have attempted to infer personality traits through behavioral features (e.g., reaction time, risk preference), but most of these experiments have been conducted in controlled environments, lacking validation in real-world, high-stakes adversarial contexts. Recent advancements in artificial intelligence have enabled machine learning methods for personality prediction, and several studies have explored ways to identify personality traits from social media data (Serrano-Guerrero et al., 2024; Tadesse et al., 2018). However, these methods typically rely on linguistic data, which may not be available in confrontational situations. In such contexts, decision-makers often lack direct linguistic information about their opponents, limiting the applicability of these approaches.

This study aims to bridge the gap by introducing a novel framework that leverages behavioral data from wargame-based adversarial simulations to infer the neuroticism of an opponent—not through players’ conscious awareness, but via machine learning models capable of identifying latent behavioral patterns that correlate with self-reported personality traits. Specifically, we examine whether behavioral features extracted from real-time, high-pressure gameplay scenarios correlate with the opponent’s self-reported neuroticism scores, and whether machine learning models trained on these features can accurately predict those scores. These patterns, such as unit preferences or suppression behaviors under stress, are often too subtle to be consciously recognized or interpreted by human players in real time. By simulating adversarial interactions on the Wargame Platform—which combines environmental controllability with intense decision-making pressures—we are able to extract psychologically meaningful behavioral indicators that are difficult to capture in traditional experimental settings. Ultimately, this approach offers a non-intrusive, ecologically valid, and scalable tool for psychological assessment in high-stakes environments, enhancing intelligence analysis and providing a strategic advantage in adversarial contexts.

## 2. Materials and Methods

### 2.1. Participants

To ensure participants’ understanding of the game and account for the unique characteristics of the Wargame Platform, participants were selected through coordination with the MiaoSuan Wargame Platform. The selection process was based on the following criteria:

- Game knowledge: Participants were required to demonstrate a comprehensive understanding of the game rules and strategies to ensure effective participation in the wargame. Participants’ familiarity with the wargame was assessed via a pre-match questionnaire that captured self-reported indicators of experience and perceived competence. Specifically, the questionnaire included items on participants’ years of experience with wargames (i.e., length of engagement), frequency of gameplay, and a self-rating of their strategic proficiency.
- Questionnaire completion: Upon registering for a match, players were asked to complete a detailed questionnaire provided by the platform, which collected basic demographic information and assessed their familiarity with the game.
- Game data integrity: Only players with complete game data were included in the study. Specifically, complete data referred to game logs that contained full match history, unit deployment sequences, and outcome records for all rounds. Incomplete records were excluded to maintain the reliability and validity of the research findings.

After registration, participants completed the platform’s questionnaire, which included fields for player ID and basic demographic details. Once the player ID was obtained, relevant game data was retrieved and downloaded from the backend. Given the wargame’s appeal to a strategic audience, particularly males with an interest in historical and military themes, the gender distribution in our study mirrors this trend. This was considered to ensure that the sample accurately represented the platform’s user base (Micheletti et al., 2018).

### 2.2. Neuroticism Measurement

To assess participants’ neuroticism, we used the neuroticism dimension of the Big Five Inventory (John et al., 1991). This scale consists of 8 items, which participants rate on a 5-point Likert scale ranging from 1 (strongly disagree) to 5 (strongly agree). The neuroticism score is calculated by averaging the scores of the 8 items, with higher scores indicating higher levels of neuroticism. In our sample, the internal consistency reliability (Cronbach’s alpha) of the neuroticism scale was 0.71, indicating acceptable internal reliability. The Big Five Inventory exhibits strong psychometric properties, with internal consistency reliability estimates ranging from 0.73 to 0.83 across different subscales and test–retest reliability estimates ranging from 0.80 to 0.90. The BFI has previously demonstrated strong psychometric properties. Future research may benefit from adopting more comprehensive neuroticism assessments to improve the accuracy and reliability of predictions.

### 2.3. The MiaoSuan Wargame Platform for Behavioral Data Collection

In this study, we utilized the MiaoSuan Wargame Platform, developed by the Intelligent System and Engineering Research Center of the Chinese Academy of Sciences Automation Institute. This human–machine platform is designed for multi-agent, incomplete information games, providing a dynamic and realistic environment for strategic decision-making (Xu et al., 2022). The platform’s primary goal is to simulate real-world military and strategic scenarios, enabling researchers to analyze human decision-making under conditions of uncertainty and competition. For this research, we employed the Human–Human Competition mode, which enables direct player competition, closely replicating real-life strategic interactions.

### 2.4. Procedure

**Figure 1:**
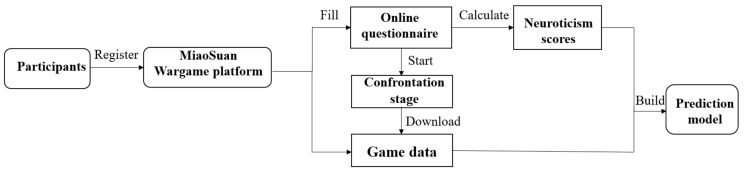
Neuroticism Assessment Process on the MiaoSuan Wargame Platform.

Considering the potential differences in participant states between real matches and training sessions, as well as the distinctive nature of the wargame, with its relatively small player population, we chose to use game data from regularly conducted matches on the MiaoSuan Wargame Platform for this study. Upon registering for matches, players were provided with a link to voluntarily complete a survey. The survey included their player ID, game level, demographic details (e.g., gender), and a neuroticism assessment. After the games were completed, we collected relevant game data by cross-referencing the obtained questionnaire information. The research process is illustrated in Figure 1.

### 2.5. Behavior Feature Extraction

After successfully parsing the game files of 167 participants, we extracted detailed gameplay data for each participant, ultimately identifying 448 distinct gameplay behavior features. Following a thorough filtering process, we excluded features that had zero values across all participants. These features lacked discriminative power and were therefore removed to ensure the resulting feature matrix contained only informative and analytically useful variables. The final set comprised 343 valid behavior features, encompassing a wide range of in-game actions that provide valuable data for the subsequent analysis of combat outcomes and damage-related behaviors.

To systematically quantify the features related to combat results and damage, we categorized these features into four major types: Attack Frequency, Suppression Frequency, Ineffective Attack Frequency, and Average Damage. This categorization was based on the official rules and mechanics of the wargame platform, which define the nature and outcomes of different unit actions. Each behavior feature was assigned to a category according to its operational role within the game system, ensuring that the classification reflected objective and rule-based distinctions rather than subjective interpretation. To ensure consistency and comparability in feature processing, we standardized the calculations using the following formulas (see Table 1 for specific calculations):

- Attack Frequency: This measures the number of attacks performed by a player within a specified time frame, reflecting their inclination towards active offensive strategies.
- Suppression Frequency: This calculates the frequency with which a player exerts suppression on the opponent during combat, reflecting their control over the engagement.
- Ineffective Attack Frequency: This measures the frequency of attacks that fail to hit their target, reflecting the player’s accuracy and resource management abilities.
- Average Damage: This represents the average damage dealt by the player per attack, serving as an indicator of their attack efficiency and overall combat contribution.

These features comprehensively reflect the player’s decision-making patterns, engagement strategies, and risk management in combat scenarios. During the feature extraction process, particular attention was paid to the influence of different weapon choices on combat outcomes, including metrics such as Kills, Losses, Suppression Frequency, Ineffective Attack Frequency, and Average Damage for each weapon. Through the analysis of these behavioral features, we gain deeper insights into the combat behaviors and strategic choices of players. For instance, some players may exhibit a tendency for frequent attacks, while others may focus more on suppression and strategic damage distribution. Analyzing these data allows us to uncover the decision-making trends of players during combat, which can inform the optimization of game design and player feedback mechanisms.

### 2.6. Feature Selection

Feature selection is a critical step in improving the performance and efficiency of machine learning models. The primary goal of feature selection is to eliminate irrelevant or redundant features, which reduces model complexity, improves accuracy, and decreases runtime. This process simplifies the model, enhances the understanding of the underlying data generation process, and ensures that only the most relevant features are included for analysis. Feature selection is grounded in the principles of dimensionality reduction, accuracy enhancement, and model interpretability. By reducing the number of features, we not only minimize model complexity but also shorten training times and reduce the risk of overfitting. Furthermore, selecting features with the highest relevance to the target variable improves predictive accuracy, while a simpler model structure is more interpretable, facilitating both analysis and communication of results. In this study, feature selection was performed using a greedy algorithm implemented in Python 3. This algorithm iteratively selects the most optimal feature at each step, progressively improving the model’s performance until no further improvements can be made. This approach ensures that only the most relevant features are retained, which enhances the efficiency and effectiveness of the prediction process. Due to the large number of features to choose from, please refer to Table A1 for more details. The table contains feature names and types to ensure transparency in the modeling process.

### 2.7. Exploratory Correlation Analysis Between Features and Neuroticism

To preliminarily assess the psychological relevance of the selected behavioral features, we conducted an exploratory correlation analysis. This analysis aimed to determine whether any individual feature extracted from the wargame logs exhibited a statistically meaningful linear association with the opponent’s neuroticism level. Specifically, Pearson correlation coefficients were calculated between each of the 343 valid behavioral features and the self-reported neuroticism scores of the opponents.

### 2.8. Regression Model for Predicting Neuroticism

Once feature selection was completed, several machine learning algorithms were employed to predict neuroticism. The selection of these models was based on their ability to handle complex relationships within the data, their suitability for the task, and their overall predictive performance (Doan & Kalita, 2015; Ray, 2019). The models included Linear Regression, Light Gradient Boosting Machine Regressor (LGBMRegressor), Linear Support Vector Regressor (LinearSVR), RandomForestRegressor, and Random Forests in XGBoost (XGBRFRegressor). A brief justification for the selection of each model is provided in Table 2.

To evaluate the generalization ability of each model, we employed 3-fold cross-validation (Koul et al., 2018). This technique divides the dataset into three subsets, using two subsets for training and the remaining subset for testing. This process is repeated three times, with each subset serving as the test set once, thereby ensuring the model’s performance is evaluated across different data splits. The results of these evaluations were then used to identify the most effective and generalizable model for predicting neuroticism, ultimately contributing to the model’s overall accuracy and interpretability. The models were evaluated using Pearson’s correlation coefficient (r), which measures the strength and direction of the linear relationship between predicted and actual values, and the coefficient of determination (R^2^), which indicates how well the independent variables explain the variance in the dependent variable. Using these metrics, we assessed each model’s ability to predict neuroticism and determined the most accurate and reliable model.

### 2.9. Reliability Analysis

To evaluate the reliability of the models tested for predicting neuroticism, we employed split-half reliability, a method commonly used in psychological research to assess the internal consistency of a test. Given the unique data collection approach in this study, we applied split-half reliability to assess the models’ reliability. Specifically, we selected 29 participants who had participated in more than 8 matches from the statistics of all player matches. This subset was used as the split-half group for evaluation.

## 3. Results

### 3.1. Descriptive and Correlational Analyses

After excluding users with incomplete game data, a total of 167 valid participants were included in the study (male = 89.8%, M_age = 22.78). Detailed information is provided in Table 3. Participants played between 1 and 18 matches, with an average of 4.48 matches (SD = 3.59). Descriptive statistics indicated that self-reported neuroticism scores ranged from 1.00 to 3.88, with a mean of 2.32 (SD = 0.67).

To further evaluate the psychological relevance of the behavioral features used for neuroticism prediction, we conducted an exploratory correlation analysis. Specifically, Pearson correlation coefficients were calculated between each of the 343 extracted features and the self-reported neuroticism scores of the opponent. Among them, 22 features showed statistically significant correlations (*p* < 0.05).

These features capture interactions between specific unit types under combat conditions, reflecting how behavioral tendencies manifest in tactical decision-making—for instance, how frequently Artillery units suppress Medium Tanks, or how often UAVs are damaged by Anti-Aircraft Guns. Such interactions may reflect underlying psychological tendencies, including caution, aggression, or susceptibility to stress. A complete list of these 22 significant features and their correlation coefficients is provided in Table A2.

### 3.2. The Performance of Predicting Neuroticism

**Figure 2:**
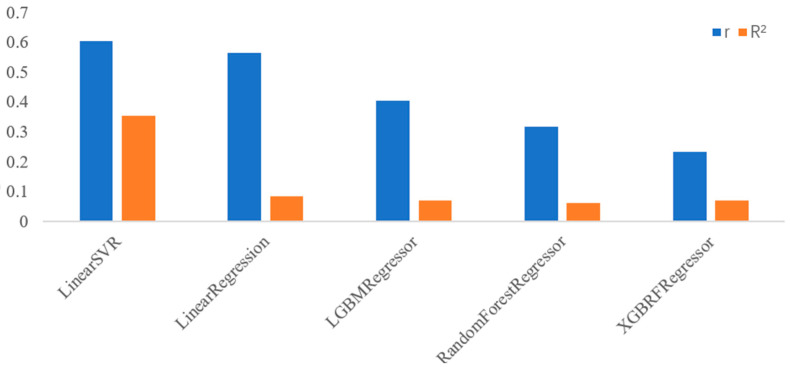
Visual comparison of the performance of regression models with 3-fold cross-validation.

After feature selection, we assessed the performance of the different models by calculating the correlation coefficients (r) and R-squared (R^2^) values for each model. Table 4 and Figure 2 present these metrics, highlighting the effectiveness of each model in predicting neuroticism scores based on the selected features. Among the models tested, LinearSVR demonstrated the best performance, achieving a correlation coefficient (r) of 0.606, indicating a moderate-to-strong positive linear relationship between predicted and actual neuroticism scores. Additionally, the R-squared (R^2^) value of 0.354 suggests that approximately 35.4% of the variability in neuroticism scores can be explained by the model. These results demonstrate the efficacy of LinearSVR in capturing the relationship between game behavior features and neuroticism.

### 3.3. The Reliability of Regression Models

In this study, cross-validation tests were carried out on all the predictive models of neuroticism produced by different algorithms to select models that excelled in both validity and reliability. It is important to note that the most valid model is not necessarily the most reliable.

Participants in the split samples were removed from the original dataset, resulting in a new training sample of 138 subjects for model reconstruction. Feature selection and model training were then conducted using the same methods as previously employed, leading to the construction of a prediction model based on the new training sample. For the remaining 29 participants, their game data were divided into two halves to create two sets of features that were used as input for predicting their neuroticism within the split sample. The reconstructed model calculated neuroticism scores for each participant based on these two inputs, representing their two measurements. The correlation coefficient between these two measurements reflects the split-half reliability of the model.

We also assessed the reliability of the model that showed the best overall performance. Table 5 shows the split-half reliability and performance of the LinearSVR regression model. Considering the reliability and validity of the model, we selected Model 1 as our optimal model (validity = 0.606, R^2^ = 0.354, split-half reliability = 0.516).

## 4. Discussion

### 4.1. The Feasibility of Predicting Opponent’s Neuroticism from Behavior in Wargame 

This study aims to evaluate the feasibility of predicting an opponent’s neuroticism through a player’s behavioral data collected from adversarial wargame simulations. While traditional assessments of personality traits typically rely on individuals’ self-reports, such information is not accessible in real-time competitive settings. Therefore, we explored whether machine learning models could infer the neuroticism level of a player’s opponent—based solely on the player’s own strategic decisions, actions, and behavioral patterns during gameplay. To this end, we used behavioral features extracted from wargame logs to train and evaluate multiple predictive models. Among them, the LinearSVR model demonstrated the strongest performance, achieving a correlation coefficient of 0.606 between predicted and self-reported neuroticism scores, an R-squared value of 0.354, and a split-half reliability of 0.516. These results suggest that the opponent’s psychological profile—particularly neuroticism—can be inferred with moderate accuracy from behavioral patterns observed in high-pressure, adversarial decision-making contexts.

### 4.2. Psychological Interpretation of Predictive Features

To deepen our understanding of the neuroticism prediction model, we conducted a feature-level analysis aimed at interpreting how specific behavioral variables contributed to model performance. While the model itself was trained in a data-driven fashion without pre-assumed psychological mappings, we retrospectively examined whether the most predictive features could be meaningfully associated with existing psychological theories on neuroticism. Specifically, we focused on weapon choices and unit interaction patterns, which were categorized into three strategic groups: infantry/support equipment, ground combat vehicles, and air combat equipment (see Table A3).

The following section will explore how each weapon category contributes to neuroticism prediction based on these classifications. The full list of weapon types and their assigned categories is summarized in Table A3, which supports the feature-level interpretation of behavioral tendencies related to neuroticism.

To further validate the psychological relevance of these features, we also conducted an exploratory correlation analysis. Among the 343 extracted behavioral features, 22 showed statistically significant correlations with self-reported neuroticism. While this number may appear limited, it reflects the well-documented challenge in psychological modeling, where individual behavioral indicators often have weak marginal effects. Importantly, machine learning models can capture multivariate and nonlinear interactions among features that are not evident in isolated correlations. Thus, even though only a subset of features reaches statistical significance in bivariate analyses, their collective contribution enables the model to reliably predict neuroticism. This observation is consistent with prior research in computational personality inference, where the interpretive power lies not in any single feature, but in their joint distribution.

The heavy tank is the only vehicular unit in the game that is permitted to attack while in motion. Its oriented sprint attack behavior can be perceived as a risky action symbol, a trait that corresponds to the impulsiveness that highly neurotic individuals may display in decision-making. In a stressful military tactical environment, this impulsive behavior may represent a coping mechanism for stress, although it may not always result in an optimal tactical outcome. Individuals with high levels of neuroticism tend to experience negative emotions more readily and are more likely to adopt extreme coping measures when confronted with challenging situations (Zhu et al., 2023). This psychological trait may lead to the selection of tanks for attacking as a tactic to attempt to control the situation and cope with stress. This preference reflects the mindset of highly neurotic individuals in strategic simulations, who respond to stressful or adverse situations by taking aggressive measures in games. This is analogous to strategic choices that are high-risk but may bring high rewards. In the actual battlefield, such behavior may be perceived as highly risky, but the consequences are more limited in the game environment, which may motivate highly neurotic players to choose the strategy more frequently. Previous research has shown that individuals with higher levels of neuroticism exhibit a range of negative emotions and behaviors, including irritability, anger, sadness, anxiety, worry, hostility, self-consciousness, and vulnerability (Costa & McCrae, 1992; Goldberg, 1993). Moreover, studies have found that those with high neuroticism are inclined to act impulsively when faced with stress (Byrne et al., 2015; Cyders & Smith, 2008; Gunthert et al., 1999). Consequently, these individuals are more likely to rely on emotion-focused coping strategies rather than problem-focused ones to alleviate stress (Watson & Hubbard, 1996). In addition, they tend to resort to inefficient avoidance strategies to deal with stress (Bolger, 1990). Furthermore, individuals with high levels of neuroticism are prone to engaging in risky behaviors in situations with high potential rewards and losses (Bornovalova et al., 2009). Consequently, the incorporation of tanks as a strategic weapon within the game holds the potential to indicate the degree of neuroticism displayed by the players.

Infantry squads and artillery represent the types of units that require in-depth strategic planning and positioning on the battlefield. These units are highly valued for their long observation range, ease of concealment, and ability to provide accurate long-range fire support. In game strategy, the use of infantry squads and artillery to occupy and maintain objective points usually indicates that the player is taking an easily defended position. Due to the limited mobility of these units, their deployment and utilization necessitate meticulous strategic planning and forward-thinking on the part of the player. The combat vehicles are not only highly mobile but may also be deployed in infantry squads, thus combining the advantages of vehicle and personnel counters. Consequently, the player tends to exercise caution with this weapon, as the slightest miscalculation before the infantry have disembarked can result in an attack and potentially disastrous consequences. Prior investigations have revealed that individuals with low levels of neuroticism, characterized as emotionally stable individuals, demonstrate traits such as composure, objectivity, and meticulousness in decision-making (Mount et al., 1994). Further studies have indicated that emotionally stable individuals tend to exhibit higher levels of rationality and decisiveness in their decision-making processes (Gohm, 2003; Milyavskaya et al., 2015). Moreover, they display increased efficiency and effectiveness in their actions (Komarraju et al., 2011). Additionally, individuals with low levels of neuroticism exhibit a composed, rational, and positive emotional response when confronted with challenges (Suls et al., 1998). Therefore, these weapon types can also, to some extent, reflect the players’ level of neuroticism.

Our study integrates the behavioral characteristics of players who utilize infantry squads and tanks, along with the behavioral traits typical of individuals with low levels of neuroticism. Through this analysis, we observed significant similarities in the behaviors exhibited by these players, indicating that these weapon types can serve as a partial reflection of the player’s level of neuroticism.

This article does not include a discussion on aerial combat equipment. We propose that these weapon types may also be influenced by neurotic predictors that have yet to be explored. However, since we have already conducted a thorough examination of the relationship between other weapon types and neuroticism, as well as elucidated the effectiveness of the LinearSVR model in predicting individual neuroticism levels, future research can delve into exploring the association between these weapon types and neuroticism. Furthermore, researchers can conduct comprehensive and in-depth investigations into the psychological impact, behavioral characteristics, and usage scenarios of these weapons, in order to gain a deeper understanding of their influence on individual neuroticism.

### 4.3. The Possible Implications of Competitive Scenarios

This study highlights the potential of identifying an individual’s level of neuroticism through their behavior in competitive scenarios. In other words, there is a correlation between situations presented in competitive environments and those found in real life, with human behavior displaying certain parallels in both virtual and physical realms (Bayraktar & Amca, 2012; Gentile et al., 2004; Yee et al., 2011). As a result, evaluating behavior within competitive scenarios offers the advantage of reducing the time needed for participants to complete questionnaires while providing access to valuable match data. This approach presents a novel method for measuring psychological traits, eliminating the need for expensive and invasive procedures. By harnessing information on a participant’s competitive behavior, predicting their level of neuroticism becomes feasible through efficient, cost-effective, and non-intrusive means. Specifically, observable behavioral features—such as unit preferences, response timing, and risk-taking tendencies—are extracted from gameplay logs and used as input variables. These features are mapped to self-reported neuroticism scores, which serve as training labels for the model. Thus, the model learns associations between behavioral patterns and individual differences in neuroticism.

Moreover, considering that our study focuses on how behavior in wargame scenarios can predict a participant’s level of neuroticism, it can also offer valuable support to the artificial intelligence systems utilized in wargame platforms. By acquiring information on the neuroticism of opponents, an AI system can predict future actions of participants while also assisting in the selection of combat commanders.

These results also offer insights into the adaptive behaviors individuals adopt during competitive interactions. Players with high levels of neuroticism tend to exhibit more impulsive and risk-prone strategies, which may serve as maladaptive coping mechanisms under pressure (Cyders & Smith, 2008; Gunthert et al., 1999). In contrast, emotionally stable individuals are more likely to engage in measured, strategic actions that reflect a higher degree of cognitive control (Mount et al., 1994; Gohm, 2003; Milyavskaya et al., 2015; Komarraju et al., 2011). Such behavioral distinctions highlight how personality traits influence the ways in which individuals adapt to high-stakes environments. The observed relationships suggest that neuroticism not only shapes how players respond to external threats but also how they regulate their internal emotional states during adversarial decision-making (Watson & Hubbard, 1996; Bolger, 1990). As a result, the predictive model captures not merely static behavior, but dynamic patterns of adaptation that reflect deeper psychological processes.

### 4.4. Limitations and Future Work

One limitation of this study is the relatively small sample size, primarily due to the recent development of the specific wargame, which resulted in a limited pool of registered players. Additionally, certain instances of invalid data had to be excluded during the gameplay data collection phase due to uncontrollable factors. Despite the small sample size, the findings provide valuable insights for future research aimed at predicting neuroticism through gameplay behavior. This study revealed a moderate correlation between the actual and predicted values. Future research could focus on collecting additional samples to further investigate gameplay attributes and enhance the model’s predictive performance and reliability.

Another important limitation concerns the use of self-report data to measure the opponent’s neuroticism. While our study aims to move beyond traditional personality assessments by developing machine learning models that infer neuroticism from observable gameplay behavior, we necessarily relied on participants’ self-reported neuroticism scores as ground-truth labels during the training phase. This may appear contradictory to our critique of self-reports; however, in real-world adversarial scenarios, it is practically impossible to obtain such self-assessments from one’s opponent. Individuals in competitive or conflict situations are unlikely to disclose sensitive psychological traits, either deliberately or due to lack of awareness. Therefore, the use of self-reports in this study serves only as a proxy to enable model development, not as a proposed long-term solution for personality assessment. Future studies could incorporate alternative sources of personality evaluation, such as observer ratings or physiological markers, to reduce reliance on self-report and improve ecological validity.

## 5. Conclusions

In summary, this study demonstrated the feasibility of using behavior in a wargame to predict neuroticism, offering a significant advantage in competitive scenarios. The predictive performance supports the potential utility of behavior in a wargame, while the reliability analyses underscore its robustness. This research not only presents a novel approach to psychological assessment but also deepens strategic understanding in competitive environments.

## Figures and Tables

**Table 1:** Feature Processing Rules for Battle Outcomes and Damage Frequencies.

Feature Type	Calculation	Naming Rule	Battle Outcome Meaning	Battle Damage Meaning
Attack Frequency	*α* = *N*_a_/*N*_t_	{Own Unit}_{Enemy Unit}_BattleOutcome/BattleDamage_Frequency	The frequency with which this unit initiates an attack against the enemy unit.	The frequency with which this unit suffers self-inflicted damage when attacking the enemy unit.
Suppression Frequency	*β* = *N*_0_/*N*_a_	{Own Unit}_{Enemy Unit}_BattleOutcome/BattleDamage_SuppressionFrequency	The frequency with which this unit suppresses the enemy during an attack.	The frequency with which this unit gets countered or disrupted when attacking.
Invalid Attack Frequency	*γ* = *N*_−_/*N*_a_	{Own Unit}_{Enemy Unit}_BattleOutcome/BattleDamage_InvalidFrequency	The frequency with which the unit’s attack is ineffective, resulting in no suppression or damage.	The frequency with which the unit’s attack is ineffective and causes additional self-inflicted damage.
Average Damage	*δ* = *S*_+_/*N*_a_	{Own Unit}_{Enemy Unit}_BattleOutcome/BattleDamage_AverageDamage	Average health reduction inflicted per successful attack	Average health reduction suffered per attack due to self-inflicted damage

Note: *α*: Attack Frequency; *N*_a_: Number of matches where this type of attack was executed (non-“/” battle records); *β*: Suppression Frequency; *N*_t_: Total number of matches; *N*_0_: Number of matches where the attack resulted in suppression (“0”); *γ*: Invalid Attack Frequency; *N*_−_: Number of matches where the attack was invalid (negative values); *δ*: Average Damage; *S*_+_: Sum of all positive battle outcome/battle damage values.

**Table 2:** Overview of Machine Learning Models Used for Predicting Neuroticism.

Rank	Model	Key Characteristics	Strengths
1	Linear Regression	Simple, interpretable model assuming linear relationships.	Serves as a baseline model for comparison with complex models.
2	Light Gradient Boosting Machine Regressor (LGBMRegressor)	Faster training speeds, lower memory usage, strong predictive performance.	Faster training speeds, lower memory usage, strong predictive performance.
3	Linear Support Vector Regressor (LinearSVR)	Effective for linear relationships, robust against noise, handles nonlinearity.	Robust against noise, handles nonlinearity.
4	Random Forest Regressor (RandomForestRegressor)	Ensemble method using multiple decision trees, good at capturing complex patterns and handling noise.	Captures complex patterns, handles noisy data and outliers.
5	Random Forests in XGBoost (XGBRFRegressor)	Combines the power of Random Forests with XGBoost’s boosting algorithm.	High predictive accuracy, computational efficiency.

**Table 3:** Sample Distribution in Wargame Confrontation.

Demographic Information	Cases (N = 167)
	Mean (SD)
Age (years)	22.78 (2.01)
Wargame Level (self-evaluation)	1.30 (0.65)
	Participants, n (%)
Gender	
male	150 (89.8)
female	17 (10.2)
Player Matches	
1	36 (21.6)
2	32 (19.2)
3–4	32 (19.2)
5–7	38 (22.8)
8–18	29 (17.4)

**Table 4:** The performance of the regression models with 3-fold cross-validation.

Method	r	R^2^
LinearSVR	0.606	0.354
LinearRegression	0.564	0.085
LGBMRegressor	0.406	0.069
RandomForestRegressor	0.316	0.062
XGBRFRegressor	0.234	0.069

**Table 5:** Performance and Split-Half Reliability of the Regression Models.

Method	r	R^2^	Split-Half Reliability
LinearSVR	0.606	0.354	0.516

## Data Availability

All data that support the findings of this study are included in this manuscript. Additionally, the data are available from the corresponding author upon reasonable request.

## Appendix A

**Table A1:** Remaining features after feature selection.

	Type	Features Selected
Battle Outcome	Attack Frequency	Medium Tank_Infantry Squad_BattleOutcome_Frequency, Infantry Squad_Infantry Squad_BattleOutcome_Frequency, Attack Helicopter_Attack Helicopter_BattleOutcome_Frequency, Infantry Squad_Medium Tank_BattleOutcome_Frequency, Anti-Aircraft Gun_Heavy Tank_BattleOutcome_Frequency, Artillery_Heavy Tank_BattleDamage_SuppressionFrequency
	Suppression Frequency	Infantry Squad_Heavy Tank_BattleOutcome_SuppressionFrequency, Anti-Aircraft Gun_UAV_BattleOutcome_SuppressionFrequency, Anti-Aircraft Gun_Attack Helicopter_BattleOutcome_SuppressionFrequency, Heavy Tank_Anti-Aircraft Gun_BattleOutcome_SuppressionFrequency, Heavy Tank_Attack Helicopter_BattleOutcome_SuppressionFrequency, Artillery_Heavy Tank_BattleDamage_SuppressionFrequency
	Invalid Attack Frequency	Infantry Squad_Heavy Tank_BattleOutcome_InvalidFrequency, Attack Helicopter_Heavy Tank_BattleOutcome_InvalidFrequency, Anti-Aircraft Gun_UAV_BattleOutcome_Invalid Frequency, HeavyTank_MediumTank_BattleOutcome_InvalidFrequency, Medium Tank_Heavy Tank_BattleOutcome_InvalidFrequency
	Average Damage	Artillery_Attack Helicopter_BattleOutcome_AverageDamage, HeavyTank_Infantry Squad_BattleOutcome_AverageDamage, AttackHelicopter_HeavyTank_BattleOutcome_AverageDamage
Battle Damage	Attack Frequency	Attack Helicopter_UAV_BattleDamage_Frequency, Artillery_UAV_BattleDamage_Frequency, Infantry Squad_Heavy Tank_BattleDamage_Frequency, Heavy Tank_Infantry Squad_BattleDamage_Frequency, Infantry Squad_Infantry Squad_BattleDamage_Frequency
	Suppression Frequency	Artillery_Medium Tank_BattleDamage_SuppressionFrequency, Heavy Tank_Infantry Squad_BattleDamage_SuppressionFrequency, Attack Helicopter_Anti-Aircraft Gun_BattleDamage_SuppressionFrequency, UAV_Anti-Aircraft Gun_BattleDamage_SuppressionFrequency, Anti-Aircraft Gun_Attack Helicopter_BattleDamage_SuppressionFrequency, Artillery_Heavy Tank_BattleDamage_SuppressionFrequency, Unmanned Tank_Attack Helicopter_BattleDamage_SuppressionFrequency, Artillery_Attack Helicopter_BattleDamage_SuppressionFrequency, Heavy Tank_Attack Helicopter_BattleDamage_SuppressionFrequency, Attack Helicopter_Heavy Tank_BattleDamage_SuppressionFrequency, Anti-Aircraft Gun_Anti-Aircraft Gun_BattleDamage_SuppressionFrequency, Heavy Tank_Attack Helicopter_BattleDamage_SuppressionFrequency
	Invalid Attack Frequency	Infantry Squad_Heavy Tank_BattleDamage_InvalidFrequency, Anti-Aircraft Gun_Anti-Aircraft Gun_BattleDamage_InvalidFrequency, Anti-Aircraft Gun_Heavy Tank_BattleDamage_InvalidFrequency, UAV_Anti-Aircraft Gun_BattleDamage_InvalidFrequency
	Average Damage	Unmanned Tank_Attack Helicopter_BattleDamage_AverageDamage, Heavy Tank_Attack Helicopter_BattleDamage_AverageDamage, Infantry Squad_Anti-Aircraft Gun_BattleDamage_AverageDamage, Infantry Squad_Heavy Tank_BattleDamage_AverageDamage, Infantry Squad_Unmanned Tank_BattleDamage_AverageDamage, Heavy Tank_Unmanned Tank_BattleDamage_AverageDamage

**Table A2:** Correlation between Game Behavior Features and Neuroticism Scores.

Feature	r
Feature1	−0.245 **
Feature2	0.203 **
Feature3	0.160 *
Feature4	0.160 *
Feature5	0.195 *
Feature6	0.228 **
Feature7	−0.249 **
Feature8	−0.159 *
Feature9	−0.168 **
Feature10	0.205 **
Feature11	0.205 **
Feature12	0.175 *
Feature13	0.163 *
Feature14	0.199 **
Feature15	0.163 *
Feature16	0.219 **
Feature17	0.157 *
Feature18	0.214 **
Feature19	0.153 *
Feature20	0.207 **
Feature21	0.205 **
Feature22	0.201 **

Note: The term “feature” refers to game behavior characteristics derived from wargame simulations. Specifically, feature1 represents the “Heavy Tank_Infantry Squad_Damage_Ineffective Frequency”, feature2 denotes the “Heavy Tank_Anti-Aircraft Gun_Result_Suppression Frequency”, feature3 signifies the “Heavy Armored Vehicle_Medium Armored Vehicle_Result_Average Damage”, feature4 corresponds to the “Medium Armored Vehicle_Heavy Armored Vehicle_Damage_Average Damage”, feature5 indicates the “Medium Armored Vehicle_Attack Helicopter_Result_Suppression Frequency”, feature6 refers to the “Medium Armored Vehicle_Attack Helicopter_Damage_Average Damage”, feature7 represents the “Infantry Squad_Heavy Tank_Result_Ineffective Frequency”, feature8 denotes the “Infantry Squad_Medium Armored Vehicle_Result_Suppression Frequency”, feature9 corresponds to the “Infantry Squad_Medium Armored Vehicle_Damage_Ineffective Frequency”, feature10 indicates the “Infantry Squad_Attack Helicopter_Result_Suppression Frequency”, feature11 refers to the “Infantry Squad_Attack Helicopter_Damage_Ineffective Frequency”, feature12 signifies the “Artillery_Heavy Tank_Result_Average Damage”, feature13 represents the “Artillery_Medium Armored Vehicle_Result_Frequency”, feature14 denotes the “Artillery_Medium Armored Vehicle_Result_Average Damage”, feature15 corresponds to the “Artillery_Medium Armored Vehicle_Damage_Frequency”, feature16 indicates the “Artillery_Medium Armored Vehicle_Damage_Suppression Frequency”, feature17 refers to the “Drone_Attack Helicopter_Damage_Suppression Frequency”, feature18 signifies the “Attack Helicopter_Medium Armored Vehicle_Result_Average Damage”, feature19 represents the “Attack Helicopter_Medium Armored Vehicle_Damage_Suppression Frequency”, feature20 denotes the “Attack Helicopter_Infantry Squad_Result_Ineffective Frequency”, feature21 corresponds to the “Attack Helicopter_Infantry Squad_Damage_Suppression Frequency”, and feature22 indicates the “Anti-Aircraft Gun_Heavy Tank_Damage_Suppression Frequency”. * *p* < 0.05, ** *p* < 0.01.

**Table A3:** Description of relevant weapon capabilities.

Equipment Types	Weapon Name	Weapon Capability
Ground Combat Vehicle	Heavy Combat Vehicle	Typically, powerful firepower, excellent armor, and low mobility, providing more firepower and armor than a light or medium combat vehicle at the same cost.
	Heavy Tank	Good all-around performance with good firepower, mobility, and protection
	Medium Combat Vehicle	Functions similarly to heavy combat vehicles.
	Unmanned ground vehicle	Remotely controlled, it can be used for reconnaissance or fire support and can direct the fire of the manned vehicle it is attached to. If a manned vehicle is destroyed, the unmanned vehicle is destroyed along with it.
Aerial Combat Equipment	Armed Helicopter	It has a high degree of mobility and is used in projection exercises to provide accurate fire support against enemy armored vehicles or infantry.
	Unmanned Aerial Vehicle	Higher detection ability and agility, with the narrowest field of view of any weapon, but also the narrowest field of view of being observed.
Infantry and Support Equipment	Anti-Aircraft Artillery	Specialized in countering air targets and limiting the free movement of enemy air power.
	Infantry Squad	Although its mobility is slow, it is easy to conceal and therefore has a low hit rate.
	Artillery	Its main operation is intervening aiming.
